# Monitoring the Acaricide Resistance Status and Baseline Susceptibility of Field‐Collected Populations of *Tetranychus urticae* Koch in Korea

**DOI:** 10.1002/arch.70151

**Published:** 2026-03-30

**Authors:** Md Munir Mostafiz, Hwal‐Su Hwang, Sushant Raj Sharma, Kyeong‐Yeoll Lee

**Affiliations:** ^1^ Department of Plant Medicine, College of Agriculture and Life Sciences Kyungpook National University Daegu Republic of Korea; ^2^ Teagasc, Crop Science Department, Oak Park Carlow Ireland; ^3^ Institute of Plant Medicine Kyungpook National University Daegu Republic of Korea; ^4^ Gorkha Seed and Agro Traders, Gorkha Agri Group Kathmandu Nepal

**Keywords:** strawberry production, two‐spotted spider mite, IRAC MoA, resistance ratio, resistance monitoring

## Abstract

The two‐spotted spider mite, *Tetranychus urticae* Koch, a major pest in Korean strawberry production, is known to rapidly develop resistance to acaricides. To establish *T. urticae*'s baseline susceptibility and assess its current resistance status in Korea, we tested 15 populations collected from major strawberry‐growing regions across the country in 2022 and 2023. Eight acaricides representing distinct IRAC mode‐of‐action (MoA) groups, including fenpropathrin, abamectin, etoxazole, chlorfenapyr, flufenoxuron, acequinocyl, pyridaben, and cyflumetofen, were evaluated using a standardized direct‐spray bioassay. Lethal concentration (LC_50_) values were compared to those of a laboratory strain maintained without acaricide exposure for more than 10 years to determine resistance ratios (RRs). Most field populations exhibited susceptibility (RR < 1) or low resistance (RR < 5) to fenpropathrin, etoxazole, flufenoxuron, pyridaben, and cyflumetofen. However, several populations have developed moderate resistance (RR = 5–10) to abamectin, chlorfenapyr, and acequinocyl, with one population from Jangseong exhibiting high resistance to abamectin (RR = 31.23). The geographic pattern of resistance, with regional differences, highlights localized selection pressures. These findings provide baseline susceptibility data for Korean strawberry‐infesting populations of *T. urticae* and support the design of region‐specific acaricide rotation strategies for use in integrated pest management programs.

## Summary

- Baseline susceptibility of *Tetranychus urticae* to eight acaricides was established using a long‐term laboratory strain.
- Field populations of Korea showed clear regional variation with localized resistance to abamectin, chlorfenapyr, and acequinocyl.
- Results support MoA‐based acaricide rotation and resistance management in strawberry IPM systems.

## Graphical abstract

A nationwide survey of *Tetranychus urticae* from Korean strawberry greenhouses revealed strong regional variation in acaricide susceptibility. Localized resistance to abamectin, chlorfenapyr, and acequinocyl highlights the need for region‐specific, MoA‐based resistance management strategies.

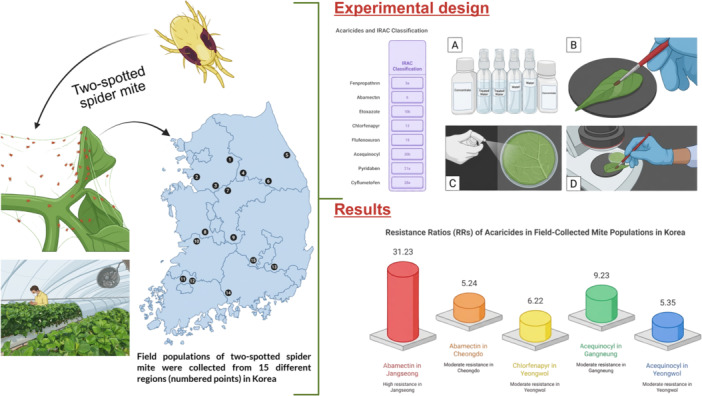

## 1 Introduction

The two‑spotted spider mite, *Tetranychus urticae* Koch (Acari: Tetranychidae), is a globally important pest of fruits, vegetables, and ornamentals and a major constraint in many protected production systems, including strawberry greenhouses (Zhang 2003; Assouguem et al. 2022). The species has high reproduction rates, a short generation period, and a wide host range, facilitating rapid population growth, and it has a well‐documented ability to develop acaricide resistance across several modes of action (Van Leeuwen and Dermauw 2016). Chemical control is still widely used, but it is becoming less effective because of this resistance, which is known to develop in multiple ways, such as mutations at the target site, increased metabolic detoxification, and physiological changes to the cuticle that reduce penetration (Van Leeuwen et al. 2006; Ilias et al. 2014; Pavlidi et al. 2015).

In Korean agriculture, strawberries are an economically important greenhouse crop, and under greenhouse conditions, sheltered environments can promote rapid increases in mite abundance, while repeated pesticide applications can select for reduced susceptibility (Ahn et al. 2021). Although resistance monitoring has been conducted for other Korean crops (e.g., apples, roses, and chives) (Song et al. 1995; Lee et al. 2003; Kang et al. 2020), comprehensive national or regional assessments of acaricide susceptibility in strawberry production remain limited. This knowledge gap is important because resistance to several widely used acaricides, including abamectin, etoxazole, pyridaben, and chlorfenapyr, has already been reported in Korean *T. urticae* populations (Lee et al. 2003; Kwon et al. 2010; Shin et al. 2021), and acaricide choice and application practices vary geographically. To effectively manage resistance, growers and advisors require up‐to‐date, regionally resolved susceptibility data. Such information enables rotation among unrelated Insecticide Resistance Action Committee (IRAC) mode‑of‑action (MoA) groups and helps avoid practices that accelerate selection pressure (Roush 1989; Kwon et al. 2015).

To address this gap*. T. urticae* populations were collected from major strawberry‐producing regions across five Korean provinces from 2022 to 2023. Using standard direct‑spray bioassays, we determined lethal concentration (LC_50_) values and calculated resistance ratios (RRs) relative to a long‑term acaricide‑free laboratory reference strain for eight acaricides representing distinct IRAC MoA groups. Our aims were to (1) provide current baseline susceptibility data for these compounds, (2) quantify resistance levels across geographically distributed field populations, and (3) determine where reduced susceptibility warrants target monitoring or management. The resulting dataset supports evidence‑based recommendations of acaricide choice and rotation for Korean strawberry production and establishes a baseline for ongoing regional resistance surveillance.

## 2 Materials and Methods

### 2.1 Mite Collection

**Figure 1:**
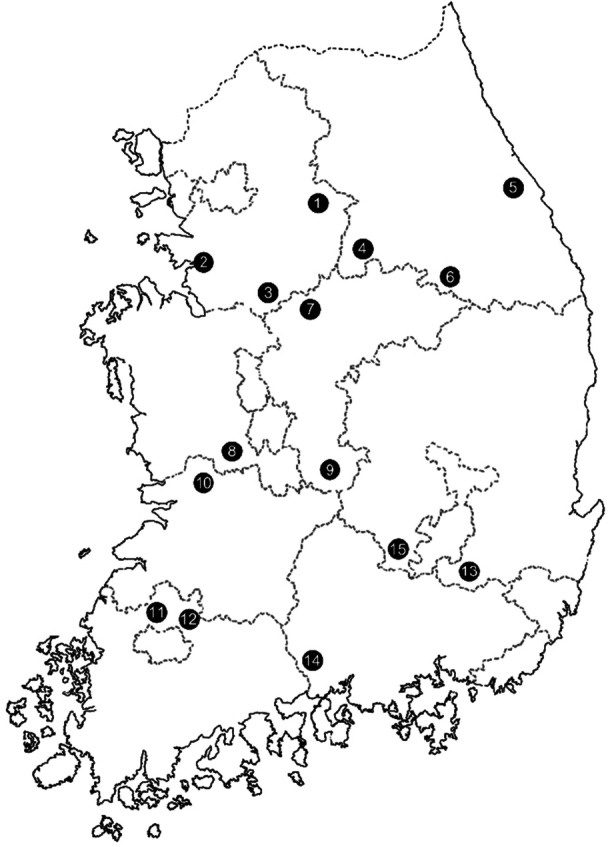
Field populations of *Tetranychus urticae* were collected from 15 different regions (numbered points) in Korea. The numbers correspond to the map nos. in Table 1.

In 2022 and 2023, we collected *T. urticae* adults from strawberry greenhouses in five provinces across Korea, targeting three regions in each province (Table 1; Figure 1). We used a susceptible strain that had been maintained in our laboratory for 10 years without any acaricide exposure. All mites were cultured on kidney bean plants (*Phaseolus vulgaris* L.) that had germinated 3 to 4 weeks before the experiment. Mites were maintained in 45 (W) × 60 (D) × 90 (H) cm insect‐proof cages under controlled conditions at 25 ± 1°C and 60 ± 10% relative humidity (RH), with a 16:8‐h light–dark cycle before use in bioassays.

**Table 1:** Collection locations and dates for the *Tetranychus urticae* populations used in this study.

Province	Map no.^a^	Region	Collection date	Latitude	Longitude
Gyeonggi‐do	1	Yangpyeong	Apr 2023	35.20589	127.8356
	2	Hwaseong	Apr 2023	37.17353	126.8668
	3	Anseong	Feb 2023	37.07644	127.4797
Gangwon‐do	4	Wonju	May 2023	37.21998	128.0885
	5	Gangneung	Mar 2023	37.85251	128.8439
	6	Yeongwol	May 2023	37.19491	128.5643
Chungcheong‐do	7	Eumseong	Apr 2023	36.92657	127.5632
	8	Nonsan	Apr 2023	36.20358	127.1102
	9	Okcheon	Mar 2023	36.36422	127.6609
Jeolla‐do	10	Iksan	May 2022	35.9185	127.0261
	11	Jangseong	Apr 2022	35.2831	126.8291
	12	Damyang	Jun 2022	35.26653	126.939
Gyeongsang‐do	13	Cheongdo	May 2022	35.6859	128.7524
	14	Hadong	May 2022	35.20589	127.8356
	15	Goryeong	May 2022	35.84618	128.4137

^a^ Map nos. correspond to those of the collection points in Figure 1.

### 2.2 Acaricides

Eight commercial acaricides, each containing a single active ingredient with a different IRAC MoA, were used in the bioassays. The active ingredients were fenpropathrin (Group 3a), abamectin (Group 6), etoxazole (Group 10b), chlorfenapyr (Group 13), flufenoxuron (Group 15), acequinocyl (Group 20b), pyridaben (Group 21a), and cyflumetofen (Group 25a). The details of each acaricide and their recommended field rates are provided in Table 2.

**Table 2:** Details of the acaricides tested against *Tetranychus urticae* in this study.

Acaricides	IRAC classification	Mode of action group	Formulation^a^	Field rate (ppm)
Fenpropathrin	3a	Pyrethroids	1 g/L EC	50
Abamectin	6	Avermectins	1 g/L EC	6.03
Etoxazole	10b	Diphenyloxazoline	0.25 g/L SC	25
Chlorfenapyr	13	Pyrrole	0.65 g/L EC	32.5
Flufenoxuron	15	Benzoylurea	1 g/L DC	50
Acequinocyl	20b	Quinoline	1 g/L SC	150
Pyridaben	21a	Pyridazinone	1 g/L EW	100
Cyflumetofen	25a	Benzoylacetonitrile	1 g/L DC	100

^a^ Abbreviations include DC, dispersible concentrate; EC, emulsifiable concentrate; EW, emulsions in water; SC, suspension concentrate.

### 2.3 Acaricide Susceptibility Bioassays for *T. urticae* Adults and Larvae

We evaluated the susceptibility of *T. urticae* to the selected acaricides using a direct spray application technique with either adult insects or larvae, depending on the insecticide's intended target. First, a pilot bioassay was conducted following the method of Shin et al. (2021) to assess the application technique. The results confirmed that direct spray applications elicited a clear dose response and that the timing of mortality assessments was appropriate. For baseline susceptibility and resistance testing, we prepared five or six concentrations of each acaricide using serial dilution in water, depending on the population and preliminary results. Test concentrations centered around the manufacturer‐recommended field rate, typically consisting of two lower and two higher levels; a sixth concentration was included when preliminary range‐finding suggested it was necessary to fully characterize the dose–response relationship.

Fenpropathrin, abamectin, chlorfenapyr, acequinocyl, pyridaben, and cyflumetofen were tested on *T. urticae* adults. Forty millimeter‐diameter kidney bean leaf discs were cut from bean leaves and placed, abaxial side up, on a bed of 1% agar in 50 mm Petri dishes, with one disc per dish. The agar served as a source of moisture, preventing the leaf disc from drying out. A 3.2‐mm hole was made in each Petri dish lid, and the lid was covered with nylon mesh to allow ventilation. Using a fine brush, 20 < 3‐day‐old *T. urticae* adult females were placed on the disc. Spray applications were performed using a 100‐mL hand‐held sprayer. Prior to the main experiment, preliminary trials established that five spray applications from a fixed distance of 20 cm consistently delivered 1 mL per leaf disc with uniform surface coverage. Control discs were sprayed with pure water. After spraying, the discs were air‐dried, lids were placed on the Petri dishes, and the dishes were transferred to a growth chamber and incubated at 25 ± 1°C and 60 ± 10% RH with a 16:8‐h light–dark photoperiod. We assessed mortality rates 72 h after treatment. Mites were considered dead if they failed to move when gently probed with a fine brush. Each concentration was tested using three to six replicate leaf discs, depending on the population and concentration, and the experiment was conducted twice, first in 2022 and again in 2023.

We evaluated the effects of etoxazole and flufenoxuron, both insect growth regulators (IGRs), on first‐instar *T. urticae* larvae using direct spray application. Single kidney bean leaves were prepared by inserting their petioles into 15 mL plastic tubes filled with water to keep the leaves fresh, and the leaves and tubes were then placed in an insect cage and incubated under the described conditions. This setup created a suitable environment for both oviposition and the subsequent bioassay. Two adult *T. urticae* females were placed on each prepared bean leaf and allowed to lay eggs. After 24 h, the adults were removed. The eggs on each leaf were then counted using a dissection microscope (Olympus, Tokyo, Japan). If more than 20 eggs were observed on a single leaf, the excess eggs were carefully removed with a fine needle to ensure a consistent number for the experiment. After 5 days, by which time all the eggs had hatched, each leaf, along with the larvae, was sprayed with five concentrations of etoxazole or flufenoxuron, or with pure water as the control, as described for the adult bioassays. Mortality was assessed 72 h after treatment using a dissection microscope. Larvae that were shriveled, discolored, and/or dried out were recorded as dead. Three to six replications (leaves) were used per concentration, and the entire experiment was performed twice, once in 2022 and once in 2023.

### 2.4 Statistical Analyses

Median LC_50_ values were calculated using a log‐probit regression analysis performed with the “PROC PROBIT” procedure in SAS version 9.4 (SAS Institute 2017), based on 72‐h mortality data for all tested acaricides. Each replicate at each concentration was entered as a separate observation rather than as a pooled mean. Therefore, the *χ*² goodness‐of‐fit degrees of freedom were based on the total number of replicates–concentration combinations, which allowed them to exceed the number of concentrations and to vary among populations. Resistance ratios were calculated for each field population as the LC_50_ value of the population divided by the LC_50_ value of the susceptible reference strain (*RR* = *LC*_*50*_ *of field samples*/*LC*_*50*_ *of susceptible samples*; Robertson and Preisler 1992). According to World Health Organization (2016) guidelines, populations were classified into four categories based on their RR: susceptible, RR < 1; low resistance, RR 1–5; moderate resistance, RR 5–10; and high resistance, RR > 10. A simple linear regression, *y *= *ax* + *b*, where *a* is the slope and *b* is the *y*‐intercept, was used to examine the relationship between the log₁₀‐transformed resistance ratio (*x*) and mortality at the recommended active‐ingredient rate (*y*) (SigmaPlot 15.0, Systat Software Inc.).

## 3 Results

### 3.1 Mortalities at the Recommended Acaricide Field Rates

**Figure 2:**
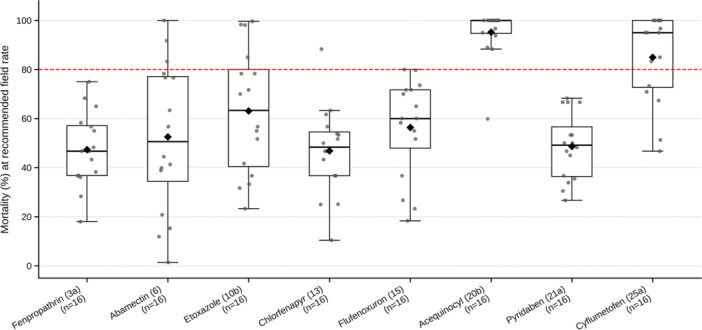
Mortality of *Tetranychus urticae* populations after 72 h of exposure to eight acaricides at the recommended field rate. Boxplots show the distribution of observed mortalities (%) across populations, with diamonds representing means and points representing individual populations. Error bars reflect sample variability. The red dashed horizontal line indicates an 80% efficacy threshold.

At the recommended field rates, mean mortalities varied substantially among the eight acaricides (Figure 2). Both etoxazole and flufenoxuron (IGRs tested on first‐instar larvae) showed intermediate activity with substantial between‐population variability (Figure 2). Etoxazole produced a mean mortality of 63.1% (range 23.3%–99.6%), exceeding the 80% threshold in only 4 of 16 populations (Figure 2). Mortality due to flufenoxuron averaged 56.4% (range 18.3%–80.0%) and reached the 80% benchmark in no populations.

The six acaricides assayed on adult females showed contrasting profiles. Acequinocyl provided the highest and most consistent control (mean 95.2%; range 59.9%–100%), with mortality in 15 of the 16 populations exceeding the 80% threshold (Figure 2). Cyflumetofen also performed strongly overall (mean 85.0%; range 46.7%–100%), although two populations experienced markedly lower mortalities (46.7% and 51.3%) (Figure 2). Abamectin showed intermediate performance (mean 52.6%), with only three populations exceeding 80% mortality (Figure 2). Fenpropathrin, chlorfenapyr, and pyridaben produced the lowest mean mortalities (47.3%, 46.8%, and 48.6%, respectively) and little evidence of consistently high control across populations (80% mortality in 0, 1, and 0 populations, respectively) (Figure 2). Overall, acequinocyl and cyflumetofen showed the most reliable efficacy across the sampled populations, whereas other compounds exhibited lower, more variable field performance.

### 3.2 LC₅₀ Values and Resistance Ratios of Field‐Collected Populations of *T. urticae* Against Eight Acaricides

**Figure 3:**
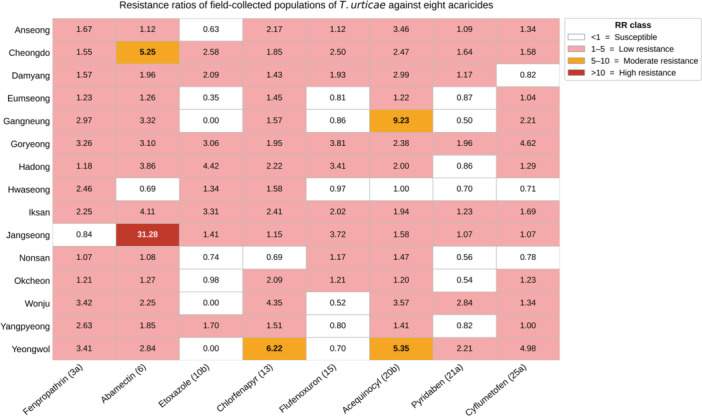
Resistance ratios of field‐collected populations of *Tetranychus urticae* from different regions of Korea against eight acaricides. Cell colors indicate resistance‐level classifications based on the resistance ratio: <1 = Susceptible, 1–5 = Low resistance, 5–10 = Moderate resistance, and >10 = High resistance.

The susceptible strain's LC_50_ values were 14.07 ppm for etoxazole and 28.18 ppm for flufenoxuron (Table 3). Across the 15 field populations, RRs ranged from ~0 to 4.42 for etoxazole and from 0.52 to 3.81 for flufenoxuron; three populations, Gangneung, Wonju, and Yeongwol, showed LC_50_ values below that of the reference strain for etoxazole (RR ≈ 0–0.01) (Figure 3). No field population's RR exceeded 5 for either IGR, indicating generally low levels of reduced susceptibility in larvae (Figure 3).

**Table 3:** LC_50_ values (with 95% confidence intervals) and regression analysis parameters for 15 Korean *Tetranychus urticae* populations and a susceptible strain against eight different acaricides after 72 h of exposure.

Acaricide	Population	*N* a	LC_50_ b	95% CI^c^	Slope (±SEM)	*X* ^2^ (*df*)^d^
Fenpropathrin (3a)	Susceptible strain	300	31.57	24.53–37.94	2.65 ± 0.37	50.62 (10)
	Anseong	300	52.7	39.67–66.65	1.65 ± 0.27	38.09 (10)
	Cheongdo	300	48.94	31.30–67.77	1.92 ± 0.39	24.33 (10)
	Damyang	300	49.5	35.66–64.46	2.34 ± 0.40	34.24 (10)
	Eumseong	300	38.92	31.85–45.83	2.75 ± 0.35	60.69 (10)
	Gangneung	762	93.85	77.22–110.94	1.51 ± 0.12	149.52 (33)
	Goryeong	360	103	74.14–145.83	1.81 ± 0.30	37.53 (13)
	Hadong	300	37.11	28.79–44.97	2.30 ± 0.32	51.95 (10)
	Hwaseong	300	77.77	62.73–97.58	1.86 ± 0.27	46.57 (10)
	Iksan	300	70.88	58.77–85.76	2.20 ± 0.29	59.3 (10)
	Jangseong	300	26.64	15.85–36.06	1.63 ± 0.29	31.19 (10)
	Nonsan	300	33.69	29.13–38.11	4.27 ± 0.57	55.69 (10)
	Okcheon	300	38.1	24.31–50.80	1.42 ± 0.27	28.52 (10)
	Wonju	455	108.09	93.26–125.81	2.21 ± 0.19	136.53 (18)
	Yangpyeong	300	82.95	66.54–105.84	1.78 ± 0.27	43.84 (10)
	Yeongwol	986	107.51	95.02–122.54	1.68 ± 0.11	225.22 (45)
Abamectin (6)	Susceptible strain	240	2.51	–e	19.05 ± 121542.1	– (7)
	Anseong	240	2.8	1.87–3.51	2.67 ± 0.51	26.95 (7)
	Cheongdo	419	13.18	11.06–16.08	2.73 ± 0.35	59.74 (15)
	Damyang	524	4.92	3.49–6.30	1.37 ± 0.17	64.67 (19)
	Eumseong	240	3.17	2.29–3.87	2.75 ± 0.49	31.07 (7)
	Gangneung	777	8.34	7.28–9.47	1.91 ± 0.14	188.6 (33)
	Goryeong	360	7.77	5.83–10.49	1.29 ± 0.26	25.61 (12)
	Hadong	547	9.68	8.82–10.61	3.35 ± 0.28	146.2 (20)
	Hwaseong	240	1.73	0.64–2.45	2.84 ± 0.76	14.14 (7)
	Iksan	486	10.32	9.15–11.62	2.85 ± 0.23	151.07 (19)
	Jangseong	476	78.52	72.91–84.1	8.91 ± 0.96	85.86 (14)
	Nonsan	240	2.72	1.94–3.30	3.34 ± 0.64	27.54 (7)
	Okcheon	240	3.2	2.49–3.76	3.45 ± 0.59	34.45 (7)
	Wonju	602	5.66	4.45–7.17	1.97 ± 0.21	84.28 (26)
	Yangpyeong	240	4.65	3.40–5.83	1.95 ± 0.41	22.76 (7)
	Yeongwol	621	7.12	6.35–7.99	2.46 ± 0.19	162.73 (26)
Etoxazole (10b)	Susceptible strain	360	14.07	10.52–17.22	2.54 ± 0.36	50.18 (13)
	Anseong	300	8.85	4.60–12.40	2.00 ± 0.38	27.35 (10)
	Cheongdo	360	36.33	24.99–50.05	1.65 ± 0.27	38.2 (13)
	Damyang	360	29.39	23.49–35.68	1.94 ± 0.22	76.03 (13)
	Eumseong	300	4.98	1.11–8.71	1.72 ± 0.43	16.19 (10)
	Gangneung	498	0.07	0.07–0.08	3.31 ± 0.28	142.88 (13)
	Goryeong	360	43.06	35.19–52.36	1.89 ± 0.21	81.26 (13)
	Hadong	360	62.13	51.34–76.05	1.94 ± 0.21	84.36 (13)
	Hwaseong	300	18.91	13.04–24.37	1.64 ± 0.28	35.61 (10)
	Iksan	360	46.52	38.13–56.43	1.92 ± 0.21	83.73 (13)
	Jangseong	360	19.78	14.39–25.06	1.74 ± 0.23	58.57 (13)
	Nonsan	300	10.36	6.42–13.58	2.31 ± 0.41	32.42 (10)
	Okcheon	300	13.79	10.24–16.87	2.60 ± 0.39	44.18 (10)
	Wonju	463	0.05	0.04–0.05	3.66 ± 0.42	74.99 (13)
	Yangpyeong	300	23.97	17.35–30.69	1.58 ± 0.27	35.1 (10)
	Yeongwol	538	0.07	0.06–0.09	3.41 ± 0.47	52.51 (13)
Chlorfenapyr (13)	Susceptible strain	360	19.34	14.35–23.75	2.40 ± 0.36	45.45 (10)
	Anseong	300	41.97	32.93–52.91	1.72 ± 0.27	41.58 (10)
	Cheongdo	360	35.77	30.90–41.12	3.20 ± 0.36	81 (10)
	Damyang	360	27.67	22.71–32.63	2.64 ± 0.33	62.36 (10)
	Eumseong	300	27.99	23.05–32.97	2.65 ± 0.33	62.98 (10)
	Gangneung	755	30.35	25.16–35.39	2.14 ± 0.18	146.16 (30)
	Goryeong	300	37.67	32.68–43.19	3.29 ± 0.36	83.15 (10)
	Hadong	360	42.88	33.87–54.32	2.80 ± 0.41	45.48 (10)
	Hwaseong	300	30.52	27.26–34.09	4.87 ± 0.55	77.96 (10)
	Iksan	360	46.52	37.87–57.69	3.20 ± 0.44	53.49 (10)
	Jangseong	360	22.33	17.66–26.67	2.62 ± 0.36	53.81 (10)
	Nonsan	300	13.32	9.01–16.38	3.45 ± 0.69	25.3 (10)
	Okcheon	300	40.46	30.39–52.46	1.50 ± 0.26	33.51 (10)
	Wonju	497	84.22	69.52–103.98	1.57 ± 0.17	88.3 (18)
	Yangpyeong	300	29.3	23.19–35.46	2.17 ± 0.29	54.3 (10)
	Yeongwol	543	120.3	90.10–178.61	2.03 ± 0.32	40.2 (22)
Flufenoxuron (15)	Susceptible strain	360	28.18	12.08–42.62	1.75 ± 0.37	21.94 (13)
	Anseong	300	31.51	21.76–40.26	1.88 ± 0.30	39.72 (10)
	Cheongdo	360	70.34	58.79–83.21	2.30 ± 0.24	93.97 (13)
	Damyang	360	54.38	42.55–66.73	1.85 ± 0.22	70.94 (13)
	Eumseong	300	22.83	15.48–28.89	2.53 ± 0.42	36.32 (10)
	Gangneung	444	24.22	13.96–40.65	1.38 ± 0.33	17.39 (13)
	Goryeong	360	107.33	82.53–141.29	2.33 ± 0.33	49.98 (13)
	Hadong	360	96.12	83.53–110.74	3.04 ± 0.28	115.19 (13)
	Hwaseong	300	27.4	19.04–34.71	2.16 ± 0.34	41.07 (10)
	Iksan	360	56.81	47.29–66.76	2.46 ± 0.26	92.17 (13)
	Jangseong	360	104.69	88.09–124.95	2.22 ± 0.22	98.33 (13)
	Nonsan	300	32.96	22.29–42.58	1.74 ± 0.29	36.66 (10)
	Okcheon	300	34.19	24.14–43.37	1.86 ± 0.29	40.77 (10)
	Wonju	434	14.7	11.08–19.50	1.12 ± 0.09	144.2 (13)
	Yangpyeong	300	22.51	13.28–30.45	1.85 ± 0.32	32.55 (10)
	Yeongwol	681	19.82	17.61–22.12	2.26 ± 0.17	179.99 (22)
Acequinocyl (20b)	Susceptible strain	360	11.59	5.09–15.57	3.40 ± 0.90	14.26 (13)
	Anseong	240	40.06	30.99–47.54	3.28 ± 0.54	37.42 (7)
	Cheongdo	300	28.61	15.83–39.34	2.01 ± 0.37	29.6 (10)
	Damyang	300	34.65	—	21.19 ± 77825.91	– (10)
	Eumseong	360	14.13	8.81–17.56	3.74 ± 0.84	19.74 (13)
	Gangneung	637	107.01	88.62–127.11	1.57 ± 0.14	129.67 (26)
	Goryeong	300	27.64	16.65–35.14	3.29 ± 0.71	21.55 (10)
	Hadong	300	23.16	9.40–30.88	3.70 ± 1.06	12.26 (10)
	Hwaseong	300	11.58	4.70–15.44	3.70 ± 1.06	12.26 (10)
	Iksan	300	22.44	10.14–31.47	2.61 ± 0.60	19.04 (10)
	Jangseong	300	18.31	6.26–28.15	2.25 ± 0.53	17.75 (10)
	Nonsan	300	17.08	—	20.82 ± 77995.61	– (10)
	Okcheon	300	13.94	8.52–17.55	3.48 ± 0.77	20.68 (10)
	Wonju	606	41.42	31.40–50.30	2.10 ± 0.25	69 (26)
	Yangpyeong	300	16.31	—	19.66 ± 78461.62	– (10)
	Yeongwol	738	62.03	45.78–75.02	3.05 ± 0.44	47.19 (30)
Pyridaben (21a)	Susceptible strain	360	104.37	90.27–119.3	3.35 ± 0.34	98.33 (13)
	Anseong	360	113.79	73.75–155.25	1.11 ± 0.20	32.41 (13)
	Cheongdo	360	171.26	139.96–207.79	1.87 ± 0.22	74.6 (13)
	Damyang	360	122.5	91.96–155.46	2.16 ± 0.29	55.47 (13)
	Eumseong	360	90.47	74.33–106.74	2.54 ± 0.28	84.02 (13)
	Gangneung	700	52.25	43.68–61.68	1.52 ± 0.13	144.07 (29)
	Goryeong	360	204.79	174.09–241.28	2.36 ± 0.24	95.85 (13)
	Hadong	360	89.81	69.19–110.18	1.97 ± 0.24	69.51 (13)
	Hwaseong	300	73.34	62.77–83.78	3.71 ± 0.47	62.81 (10)
	Iksan	360	128.23	106.00–151.98	2.24 ± 0.24	90.09 (13)
	Jangseong	360	112.15	72.52–154.28	1.73 ± 0.30	32.85 (13)
	Nonsan	360	58.88	43.99–72.47	2.38 ± 0.32	56.5 (13)
	Okcheon	360	56.14	41.36–69.48	2.39 ± 0.33	53.9 (13)
	Wonju	686	296.72	236.57–399.18	1.15 ± 0.14	68.13 (30)
	Yangpyeong	360	85.18	66.84–103.28	2.14 ± 0.25	72.34 (13)
	Yeongwol	504	230.63	182.69–301.4	1.18 ± 0.16	57.48 (22)
Cyflumetofen (25a)	Susceptible strain	360	21.77	—	19.82 ± 93086.17	– (13)
	Anseong	300	29.16	19.21–37.97	1.81 ± 0.30	37.01 (10)
	Cheongdo	360	34.46	26.41–42.11	2.27 ± 0.29	62.45 (13)
	Damyang	360	17.91	10.30–24.18	2.33 ± 0.42	30.49 (13)
	Eumseong	300	22.65	15.95–27.87	2.99 ± 0.52	32.51 (10)
	Gangneung	643	48.13	29.62–66.88	1.32 ± 0.16	71.08 (26)
	Goryeong	360	100.53	81.50–124.28	1.75 ± 0.20	73.43 (13)
	Hadong	360	28.19	23.47–32.25	4.16 ± 0.65	41.18 (13)
	Hwaseong	300	15.44	6.27–20.58	3.70 ± 1.06	12.26 (10)
	Iksan	360	36.86	26.07–47.30	1.70 ± 0.23	55.94 (13)
	Jangseong	360	23.24	15.85–29.55	2.39 ± 0.38	40.48 (13)
	Nonsan	300	17.04	8.72–21.68	4.01 ± 1.08	13.91 (10)
	Okcheon	300	26.81	20.64–32.03	3.09 ± 0.47	42.61 (10)
	Wonju	973	29.18	17.70–40.77	0.88 ± 0.11	65.47 (48)
	Yangpyeong	300	21.87	14.44–27.91	2.51 ± 0.43	34.21 (10)
	Yeongwol	474	108.34	87.18–134.64	2.83 ± 0.38	55.61 (22)

^a^ Number of mites used in the bioassay, including controls.

^b^ Median lethal concentration.

^c^ Confidence interval.

^d^ Chi‐square value and the degrees of freedom.

^e^ “–” indicates that the value was not estimable due to near‐complete mortality at all tested concentrations.

For the six acaricides assayed on adult females, resistance levels were heterogeneous among populations and compounds. Fenpropathrin, pyridaben, and cyflumetofen resistance remained in the low range across all populations (RR ≤ 4.98) (Figure 3). Chlorfenapyr's RRs were also low, except in Yeongwol (RR = 6.22). Moderate resistance to acequinocyl was observed in Gangneung (RR = 9.23) and Yeongwol (RR = 5.35). The highest resistance detected was against abamectin: the Jangseong population had an LC_50_ of 78.52 ppm (Table 3), representing the only instance of an RR > 10. Additionally, moderate abamectin resistance (RR = 5.25) was observed in Cheongdo (Figure 3).

### 3.3 Relationship Between Resistance Ratio and Mortality at the Recommended Field Rates

**Figure 4:**
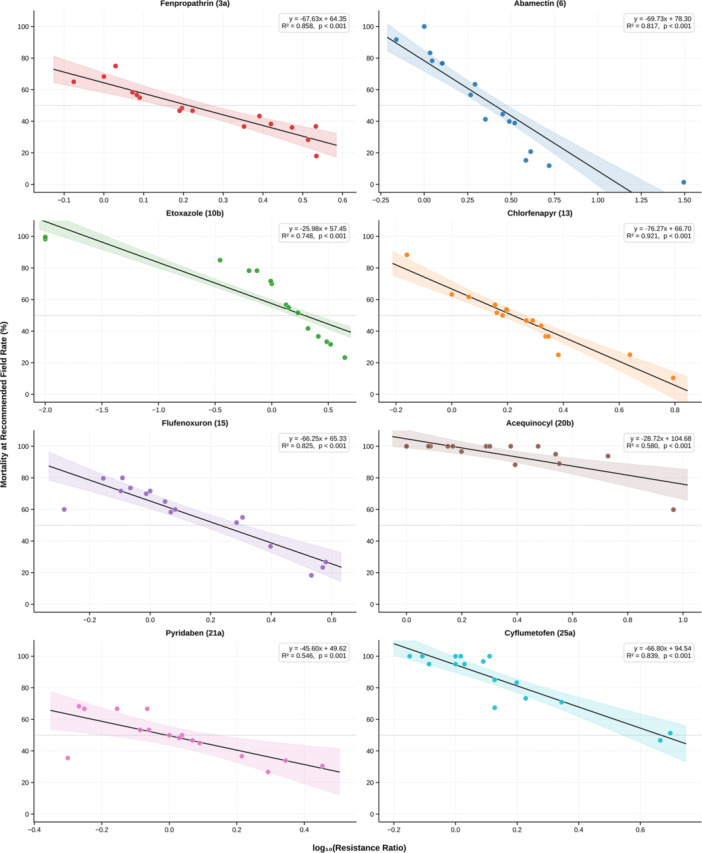
Mortality at the recommended active ingredient rate plotted against the log_10_‐transformed resistance ratio for eight acaricides across field‐collected populations. Solid lines represent fitted linear regression models and shaded areas indicate 95% confidence intervals. The regression equation, *R* ^2^ value, and *p*‐value are shown within each plot.

All eight acaricides showed significant negative relationships between log_10_(RR) and mortality at the recommended active‐ingredient rate (*p *≤ 0.001; Figure 4), i.e., mortality declined as resistance increased. Chlorfenapyr showed the strongest model fit and steepest decline (*R* ^2^ = 0.921; slope *a *= −76.3), indicating a very large drop in mortality for each tenfold rise in resistance (Figure 4). Fenpropathrin, cyflumetofen, flufenoxuron, and abamectin also exhibited strong fits (*R* ^2^ ≈ 0.82–0.86) and similarly steep negative slopes (*a *≈ −66), consistent with a rapid loss of field efficacy as resistance rises (Figure 4). By contrast, acequinocyl and pyridaben produced weaker linear relationships (*R* ^2^ = 0.58 and 0.55, respectively) and shallower slopes; acequinocyl nevertheless retained high mean mortality (~95.2%) across most populations. Etoxazole showed a relatively shallow slope (*a *= −26.0; *R* ^2^ = 0.75). Many populations with RRs < 1.0 still experienced near‑complete mortality (98%–99.6%), but as RRs increased slightly to 2–4, much lower control was achieved (23%–42%), indicating that substantial loss of efficacy can occur at moderate resistance levels despite the shallower overall slope.

## 4 Discussion

Nationwide monitoring revealed clear geographic variation in the susceptibility of *T. urticae* to the eight acaricides tested. Most populations remained susceptible or showed only low resistance, while some developed moderate to high resistance. The most concerning resistance levels were observed for abamectin, chlorfenapyr, and acequinocyl. This variation indicates that resistance management should be regionally targeted rather than based on uniform national recommendations.

Slope estimates added useful detail beyond RRs. In the Jangseong population, abamectin exhibited a very steep slope (8.91 ± 0.96) and a high RR (31.28), suggesting that resistance in this population is widespread and that selection has already run its course. In contrast, the lower slopes observed in the Goryeong (1.29 ± 0.26, RR = 3.10) and Damyang (1.37 ± 0.17, RR = 1.96) populations suggest that mixed susceptibility persists, so changing the chemistry now could still slow further selection. For chlorfenapyr, the Hwaseong population showed a steep slope (4.87 ± 0.55) despite its low RR (1.58). This combination indicates that the population warrants follow‐up, as resistance can quickly strengthen once it begins. For acequinocyl, the relatively shallow slope of the linear fit for the Gangneung population (1.57 ± 0.14), alongside its moderate resistance (RR = 9.23), suggests variation still exists within the population, so management changes may still deliver improvement.

The results broadly match earlier Korean reports but indicate clear changes over time and space. Abamectin has been used in Korea since 1993 (Pesticide Handbook 2000), and resistance has already been reported from greenhouse populations in Buyo and Chilgok (Lee et al. 2003). In this study, abamectin resistance was highest in Jangseong (RR = 31.28) and moderate in Cheongdo (RR = 5.25), suggesting that reduced susceptibility now occurs beyond previously reported areas and that higher resistance levels have developed in additional regions. For acequinocyl, moderate resistance was observed in Gangwon Province, with RRs of 9.23 at Gangneung and 5.35 at Yeongwol, making these sites a regional concentration of resistance in strawberry‐associated *T. urticae* populations. In contrast, RRs for etoxazole and pyridaben remained below 5 across all 15 strawberry populations, which contrasts with reports of high resistance emerging in other Korean cropping systems after sustained use (Koo et al. 2021). This suggests that, compared with these other systems, weaker selection pressure has occurred so far in greenhouse strawberry systems.

This study did not test cross‐resistance directly. Still, no population showed moderate or high resistance to acaricides from more than one MoA group at the same time, which supports the use of rotations across the tested groups (Sparks and Nauen 2015). Based on the current profile, abamectin (Group 6) and acequinocyl (Group 20B) should be removed from rotation where resistance is elevated. Fenpropathrin (Group 3A), etoxazole (Group 10B), flufenoxuron (Group 15), pyridaben (Group 21A), and cyflumetofen (Group 25A) remain suitable rotation options because all populations exhibited RRs below 5 for these products. Chlorfenapyr (Group 13) remains effective at many sites, but systems in Yeongwol need to take caution due to the moderate resistance encountered there. Pairing chemical rotation with non‐chemical control, including the conservation of predatory mites and adjustments in crop practices, will reduce selection pressure (Oatman et al. 1968; Oliveira et al. 2009).

The LC_50_ values and RR classes reported here provide a baseline for future comparisons. Next steps should connect these bioassay results to provincial spray histories and expand monitoring with molecular tools. Combining such methods will improve risk prediction and sharpen local guidance for Korean strawberry production.

## 5 Conclusions

This study establishes the baseline susceptibility of *T. urticae* to eight acaricides and identifies significant regional variation in resistance across Korean strawberry greenhouses. Most populations remain relatively susceptible to all MoA groups, but emerging localized resistance to abamectin, chlorfenapyr, and acequinocyl warrants targeted management. These results provide essential guidance for designing sustainable acaricide rotation programs to slow the evolution of resistance in Korea.

## Author Contributions

**Md Munir Mostafiz:** conceptualization, methodology, investigation, software, formal analysis, writing – original draft preparation, writing – reviewing and editing. **Hwal‐Su Hwang:** software, formal analysis, data curation, writing – reviewing and editing. **Sushant Raj Sharma:** formal analysis, data curation, writing – reviewing and editing. **Kyeong‐Yeoll Lee:** conceptualization, validation, resources, visualization, supervision, project administration, funding acquisition, writing – reviewing and editing.

## Conflicts of Interest

The authors declare no conflicts of interest.
